# Identification of CD8^+^ T-cell epitope from multiple myeloma-specific antigen AKAP4

**DOI:** 10.3389/fimmu.2022.927804

**Published:** 2022-07-28

**Authors:** Ning Ma, Huihui Liu, Yang Zhang, Wei Liu, Zeyin Liang, Qian Wang, Yuhua Sun, Lihong Wang, Yuan Li, Hanyun Ren, Yujun Dong

**Affiliations:** Department of Hematology, Peking University First Hospital, Beijing, China

**Keywords:** cancer-testis antigen, peptide epitope, cytotoxic T lymphocytes, multiple myeloma, dendritic cell

## Abstract

Multiple myeloma (MM) is a malignant plasma cell disorder affecting mainly the elderly population. Revolutionary progress in immunotherapy has been made recently, including monoclonal antibodies and chimeric antigen receptor T cell (CAR-T) therapies; however, the high relapse rate remains problematic. Therefore, combination therapies against different targets would be a reasonable strategy. In this study, we present a new X-chromosome encoded testis-cancer antigen (CTA) AKAP4 as a potential target for MM. AKAP4 is expressed in MM cell lines and MM primary malignant plasma cells. HLA-A*0201-restricted cytotoxic T lymphocytes (CTLs) induced by dendritic cells (DCs) transduced with an adenovirus vector encoding the full-length *AKAP4* gene were demonstrated to lyse AKAP4^+^ myeloma cells. Seven of the 12 candidate epitopes predicated by the BIMAS and SYFPEITH algorithms were able to bind HLA-A*0201 in the T2 binding assay, of which only two peptides were able to induce CTL cytotoxicity in the co-culture of peptide-loaded human mature dendritic cells and the autologous peripheral blood mononuclear cells (PBMCs) from the same HLA-A*0201 donor. The AKAP4 630–638 VLMLIQKLL was identified as the strongest CTL epitope by the human IFN-γ ELISPOT assay. Finally, the VLMLIQKLL-specific CTLs can lyse the HLA-A*0201^+^AKAP4^+^ myeloma cell line U266 *in vitro*, and inhibit tumor growth in the mice bearing U266 tumors *in vivo*. These results suggest that the VLMLIQKLL epitope could be used to develop cancer vaccine or T-cell receptor transgenic T cells (TCR-T) to kill myeloma cells.

## Introduction

The past decade witnessed great progress in multiple myeloma (MM) management, including new agents such as proteasome inhibitors (1, 2) and immuno-modulators (3, 4), immunotherapy targeting myeloma cells such as the monoclonal antibodies targeting CD38 (5) and SLAMF7 (CS-1) (6), anti-BCMA chimeric antigen receptor T (CAR-T) (7, 8), and the emerging XPO1 inhibitor (9), which all have shown to be effective to treat relapsed and refractory myeloma. However, MM remains incurable.

The high response rate of BCMA CAR-T cell therapy in refractory and relapsed myeloma patients made it a “superstar” in recent years (7, 8), but the high relapse rate after CAR-T is still the major concern: eventually the vast majority of MM patients with remission will relapse within 1 year. Given the advanced age of MM patients, it is not feasible to improve the efficacy of CAR-T therapy by sequential allogeneic hematopoietic stem cell transplantation, which had been proven to be effective in patients with acute lymphoblastic leukemia. Attempts such as sequential infusion of two CAR-T cells have been shown to be effective to improving the overall response; however, the duration of remission has yet to be evaluated due to the limited follow-up [179 days (IQR 72–295)] (10). Theoretically, combination therapies with immune effector cells against different targets would be a reasonable strategy to solve the problem of relapse. Current studies have explored the combination of natural killer (NK) cells, cytotoxic T lymphocytes (CTLs), and T-cell receptor transgenic T (TCR-T) cells. The development of TCR-T is based on the identification of naturally processed immune dominant epitopes from antigenic proteins. As shown by the Rapoport AP group, the combination of TCR-T cells targeting NY-ESO-1 peptide with autologous stem cell transplant is promising in the treatment of MM (11).

The combination of flow cytometry cell sorting and next-generation sequencing (NGS) technology has accelerated research into cancer antigens (12). One of the most impressive and promising families of cancer-specific proteins is cancer-testis antigens (CTAs) (13). CTAs are a group of testicular proteins that are aberrantly and highly expressed in tumor tissues but rarely in usual somatic tissues. Due to the limited expression and the testicular immune privilege, CTAs are considered a great target for immunotherapy (14). However, comparing with the neo-epitopes, CTA has less advantage to induce high-affinity T cells due to the central tolerance (15). MM cells express multiple CTAs, such as NY-ESO-1, MAGE, and GAGE protein families (16), and the expression levels of some proteins such as MAGE C1 are associated with disease progression and even prognosis in patients with myeloma (14). Therefore, for a long time, many researchers have used CTA-based immune methods to treat MM, and some promising results have been achieved in animal models and clinical trials (11).

A-kinase anchoring protein 4 (*AKAP4*), is a CTA gene located at Xp11.22 (17). It is speculated to be involved in intracellular signal transduction of cellular protein kinase A as a scaffolding protein. AKAP4 has high expression in MM cell lines and primary myeloma cells, but low expression in normal tissues (17). Mirandola et al. established a human MM model in NOD-RAG-1-null IL-2rg-null (NRG) mice and found that AKAP4 was not only highly expressed in myeloma cells, but also this protein, like CD138, could be used as a marker of myeloma cells to track the proliferation of malignant cells and to distinguish human myeloma cells from mouse cells (18). However, AKAP4 protein is weakly expressed on the cell surface but highly expressed in the cytoplasm. Therefore, like most CTAs, AKAP4 is not suitable to serve as the target for antibody or the conventional CAR-T therapy but could be used as a target of CTL or TCR-T.

In this study, we adopted a reverse immunological method and identified an AKAP4-derived peptide (No. 8: VLMLIQKLL, 630–638) as a naturally processed HLA-A*0201-specific epitope. CTLs induced by this peptide can effectively kill HLA-A*0201^+^AKAP4^+^ myeloma cell line *in vitro* and *in vivo*. These results indicate that this epitope might be used to develop DC vaccine and TCR-T to kill myeloma cells.

## Materials and methods

### Cell lines

The following cell lines were used in this study: the human MM cell lines U266 (HLA-A*0201^+^) and RPMI-8226 (HLA-A*0201^-^); a human myeloid leukemia monocyte cancer cell line, THP-1 (HLA-A*0201^+^); a lymphoma cell line, RAMOS (HLA-A*0201^-^); and the T2 cells (HLA-A*0201^+^), a TAP-deficient human hybrid B/T lymphoblastic cell line; cells were maintained in RPMI-1640 (U266, RPMI-8226, and T2) or DMEM (THP-1) medium (Euro Clone) supplemented with 10% fetal bovine serum (FBS, Gibco), 2 mM L-glutamine, 10 mM HEPES, 100-U/ml penicillin, and 100 U/ml streptomycin (all from Life Technologies, USA), at 37°C in a 5% CO_2_ atmosphere.

### Peptide selection and synthesis

Twelve HLA-A*0201 restricted peptides from the AKAP4 protein sequence were selected based on an integration of their score using the HLA peptide binding prediction algorithms available at BIMAS (http://www.bimas.cit.nih.gov/molbio/hla_bind/) and SYFPEITHI (http://www.syfpeithi.de/bin/mhcserver.dll/epitopeprediction.htm) websites. The CMV peptide NLVPMVATV served as positive control in the T2 stabilization assays. All peptides were synthesized by Sangon (Shanghai, China) with a purity of 98% verified by mass spectrometry analysis.

### T2 peptide binding assay

We used T2 cells to evaluate the binding and stabilization of HLA-A*0201 molecule stimulated by AKAP4-derived peptides. T2 cells were stripped in PBS for 2 h, washed, and resuspended in serum-free culture media. A total of 2 × 10^5^ cells were incubated with 3 mg/ml β2-microglobulin (Sigma-Aldrich) and 50 μM peptide in a final volume of 200 μl for 4 h at 37°C. Cells were then washed and stained with the PE-conjugated HLA-A*0201 monoclonal antibody (BioLegend) before cytometry evaluation (FACSC II, BD Biosciences). As described elsewhere (19), fluorescence intensity (FI) was calculated as the mean fluorescence intensity (MFI) of peptide-pulsed T2 cells/the MFI of T2 cells not loaded with peptide − 1.

### Generation of peptide-specific T cells from healthy donors

All the relevant health donors and patients in this study signed the informed consent. Our study complied with the Helsinki Declaration and was approved by the Ethics Committee of the First Hospital of Peking University, with the approval number of 2021-323 from the Human Ethics Committee. The CTLs were generated based on the protocol described elsewhere (19). Briefly, human mature dendritic cells (DCs) were induced using rhIL-4, rhGM-CSF, and rhTNF-α (PeproTech) from bone marrow cells of HLA-A*0201^+^ donors. DCs were then pulsed with peptide (50 µM) overnight and then cocultured with peripheral blood mononuclear cells (PBMCs) from the same donor in the presence of IL-2 (10 ng/ml). Peptide-induced CTLs were harvested after 5 days. Monoclonal antibodies used for DC and CTLs analysis included CD11c, CD80, CD86, CD3, CD4, and CD8 (BD Pharmingen).

### ELISPOT assay

Human IFN-γ precoated ELISPOT kit (DAKEWE, China) was used for ELISPOT assay according to the instructions. PBMCs were isolated from the peripheral blood of healthy donors, and some cells were used to induce the formation of DC, while others were stimulated by a mixture of IL-2 and AKAP4 peptides (all 12 peptides are mixed). On the fifth day, these cells were harvested and plated. Briefly, 2 × 10^5^ PBMCs and 5 × 10^4^ autologous DCs were co-cultured for 24 h under the stimulation of different peptides. These cells were then harvest for ELISPOT assay. The spots were developed according to the program, and each well was analyzed by a Dual color plate-reader.

### CTL killing assay

First, the target cells are labeled with CFSE (Thermo, America). One microliter of CFSE was rapidly added to 1 ml of PBS resuscitated cells (the final concentration of CFSE was 5 μM), and stained at room temperature for 10 min in the absence of light. Complete medium was then added to stop staining. Cells were centrifuged at room temperature for 5 min. The complete medium was then used to resuscitate the cells at a cell density of 1× 10^5^/ml. The cultured CTL cells and labeled target cells were laid into a 96-well concave bottom plate at a fixed effector–target ratio for 6 h. Cells were then washed twice using PBS and stained with 7-AAD antibody before examined using flow cytometry.

### *In vivo* tumor model

NTG mice were purchased from SPF Biotechnology Co. Ltd. (Beijing). and bred in the animal breeding facilities at Peking University First Hospital under specific pathogen-free conditions. The experiments were approved by the Ethics Committee of Peking University First Hospital (animal ethics approval number: 2022055). CTLs were induced by No. 8 peptide following the above-mentioned procedure. NTG mice were inoculated subcutaneously (s.c.) with 5 × 10^5^ U266 cells in the axilla (day 0). The No. 8 peptide-induced CTLs (5 × 10^6^ per mice) or PBS was injected intravenously on day 1 and day 7. Tumors were measured along two orthogonal axes (a = length, b = width) every 5 days and tumor volume was calculated by the following formula: volume = a × b × b/2.

### Statistical analyses

Wilcoxon rank sum test was used to assess difference in the data that do not meet the normality test. When comparing more than two groups, non-parametric Friedman test was first used to test the whole groups; if significant, Kruskal–Wallis rank sum test or Wilcoxon rank sum test was then used to test all possible groups. In the data that meet the normality test, unpaired *t*-test was used. When comparing more than two groups, two-way ANOVA was first used to test the whole groups; if significant, unpaired *t*-test was then used to test all possible two groups. *p-value < 0.05 was considered significant. Statistical analyses were performed using SPSS 20.0 software.

## Results

### AKAP4 was highly expressed in primary myeloma cells and the U266 cell line

**Figure 1:**
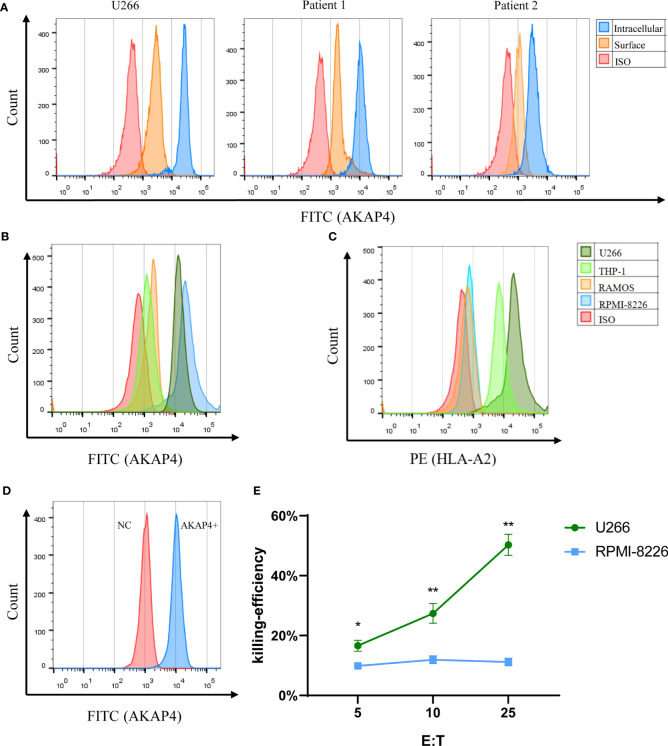
AKAP4 is expressed on primary MM cells and MM cell lines and induces specific immune responses targeting myeloma cells. **(A)** AKAP4 was expressed both intracellularly and on the cell surface in primary cells of myeloma patients (gated on the CD38^+^CD138^+^population) and in myeloma cell line U266. **(B)** Intracellular expression of AKAP4 in different cell line. **(C)** Expression of HLA-A*0201 in different cell lines after IFN- γ stimulation. **(D)** AKAP4 expression in DC cells of healthy donors after adenovirus transfection. **(E)** The killing effect of CTL on U266 cells (HLA-A 0201^+^ AKAP4^+^) induced by AKAP4 adenovirus transfection DC was higher than that of RPMI-8226 (HLA-A 0201^-^ AKAP4^+^) (summarized the results of three independent experiments). Friedman test showed the *p* < 0.05 for U266 and RPMI-8266. Then, the *p*-value was analyzed by Wilcoxon rank sum test. *p < 0.05, **p < 0.01. NC: negative control.

We examined the expression of AKAP4 by flow cytometry in common myeloma cell lines, including U266 and RPMI-8226, control cell lines THP-1 and RAMOS, and primary myeloma cells. The expression of AKAP4 was confirmed in both cell lines (U266 and RPMI-8226) and patient samples (**Figures 1A, B**). It is worth noting that the expression of AKAP4 on the cell surface was lower than that in the cytoplasm. The two control cell lines, RAMOS and THP-1, were not detected to express AKAP4 by flow cytometry. Then, we examined the expression of HLA-A*0201 on these cell lines upon INF-γ stimulation. U266 cell and THP-1 cells were HLA-A*0201 positive, while RAMOS and RPMI-8226 were HLA-A*0201 negative (**Figure 1C**).

### AKAP4 can induce effective immune response against myeloma cells

After confirming the expression of AKAP4 in the primary myeloma cells, we further explored the antigenicity of AKAP4. We cloned the full-length *AKAP4* into the adenovirus vector and transduced mature DCs. Empty adenovirus vector without *AKAP4* was used as negative controls. After 24 h, we eluted the adenovirus and co-incubated the transduced DCs with T cells from healthy donors. Successful transduction of AKAP4 was first confirmed by flow cytometry (**Figure 1D**). We then tested the killing ability of CTLs. The specific killing effect of CTLs on U266 was much higher than that on RPMI-8226 (**Figure 1E**), while CTLs induced by negative control adenovirus had no killing effect on U266 cells (**Figures S1A, B**). These results suggest that CTLs induced by AKAP4-adenovirus transduced DCs can specifically kill AKAP4-expressing U266 cells, suggesting that AKAP4 may have CTL epitopes.

### Screening high-affinity peptides using T2 affinity test and concentration gradient test

**Figure 2:**
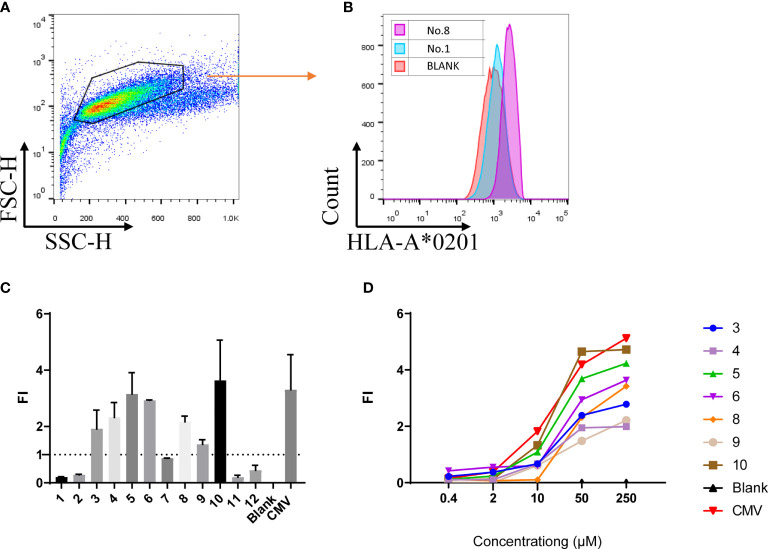
The T2 binding assay showed 7 of the 12 peptides synthesized bind the HLA-A*0201 molecule with different affinity. **(A, B)** Representative multi-parameter flow cytometry scatter diagram for T2 cell affinity detection. **(C)** FI value of 12 peptides at the concentration of 50 μM (summarized the results of three independent experiments). **(D)** FI of the 12 peptides at different concentrations (one independent experiment). Fluorescence intensity (FI) = the mean fluorescence intensity of peptide-pulsed T2 cells/the FI of T2 cells not loaded with peptide − 1. CMV: peptide derived from CMV pp65 495-503 NLVPMVATV, served as positive control; Blank: result of T2 cells not loaded with any peptide.

Given that AKAP4 successfully induced the specific CTL cytotoxicity *in vitro*, we then screened possible key peptide epitopes. First, we obtained the complete amino acid sequence of AKAP4 from NCBI, and analyzed the sequence using BIMAS and SYFPEITHI algorithms to predict the potential HLA-A*0201 restricted epitopes. Twelve peptides with the highest predicted scores were selected and synthesized for further study (**Table 1**). We then used the T2 binding test to evaluate their ability to stabilize HLA-A*0201 molecules. After overnight incubation, the cells were washed to remove unbound peptides and stained with HLA-A2-PE mAb. HLA-A2-specific peptide binding is shown as an increase in the HLA-A2 fluorescence index (FI) (**Figures 2A, B**). The experiment was repeated 3 times. The fluorescence intensity (FI) was used to indicate the affinity with HLA-A*0201. As described in the method, fluorescence intensity (FI) was calculated as the mean fluorescence intensity (MFI) of peptide-pulsed T2 cells/the MFI of T2 cells not loaded with peptide − 1; FI > 1 indicates strong affinity. **Figure 2C** illustrates the FI of 12 AKAP4 peptides along with the negative control (Blank) and the positive control (CMV peptide). Seven peptides (No. 3, No. 4, No. 5, No. 6, No. 8, No. 9, and No. 10) had FI >1, indicating good affinity with HLA-A01. Finally, we carried out concentration gradient experiments on these seven peptides. We confirmed that they all had the ability to stabilize HLA-A*0201 molecules at 50 μM. **Figure 2D** showed the concentration–affinity curve for these seven peptides with HLA molecules *in vitro*.

**Table 1:** Information of 12 peptide candidates.

No.	Amino acid sequences	Position in the protein	BIMAS score	SYFPEITHI score
AKAP4-1	T M M S D D I D W L	8	353.842	23
AKAP4-2	L L S D L Q K Y A L	114	148.896	25
AKAP4-3	Y L M N R P Q N L	167	363.588	23
AKAP4-4	S I D D L S F Y V	213	500.319	22
AKAP4-5	G L M V Y A N Q V	332	257.342	24
AKAP4-6	V L M T D S D F V	396	1179.014	22
AKAP4-7	A M L K R L V S A L	425	131.296	27
AKAP4-8	V L M L I Q K L L	630	134.369	21
AKAP4-9	K L V E S V M K L	699	705.066	29
AKAP4-10	S L Q K Q L Q A V	769	159.970	27
AKAP4-11	Q L Q A V L Q W I	773	131.975	22
AKAP4-12	Q L L D W L L A N L	845	745.355	29

### Immunogenicity of high-affinity peptides by the human IFN-γ ELISPOT assay

**Figure 3:**
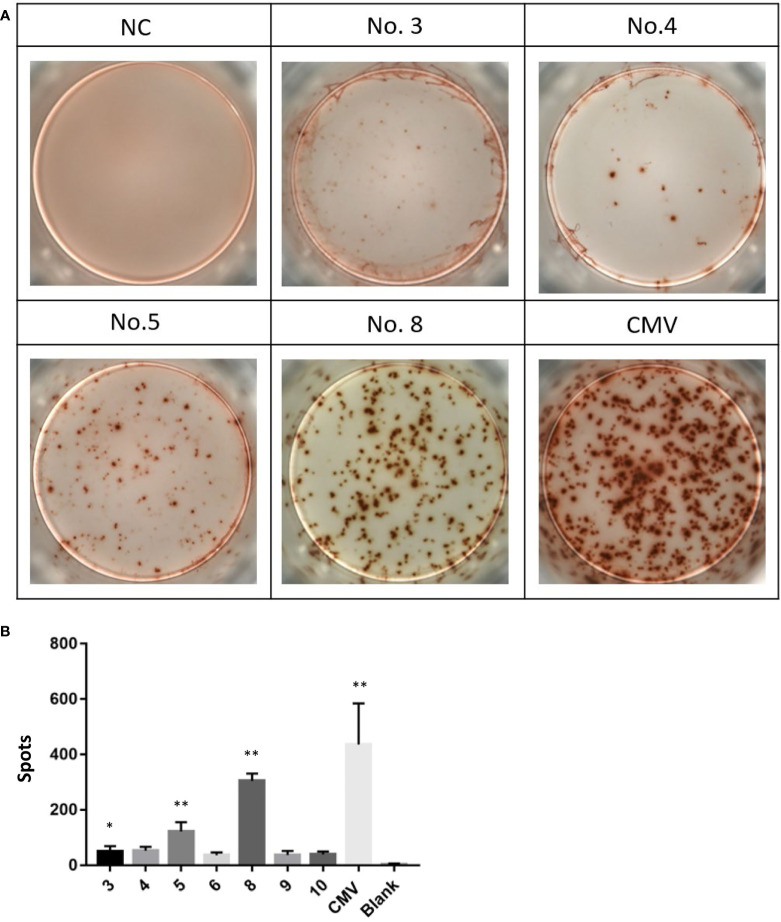
Peptides No. 5 and No. 8 of AKAP4 protein can induce T cells to secrete IFN-γ in vitro.**(A)** Images of the ELISPOT assay. NC: Negative control, No. 3, 4, 5, and 8: Peptide No. 3, Peptide No. 4, Peptide No. 5, and Peptide No. 8; CMV: CMV peptide (positive control). **(B)** The score of Peptide No. 5 and Peptide No. 8 was higher than NC in ELISPOT assay (summarized the results of three independent experiments). Kruskal–Wallis rank sum was performed to assess the significance in **(B)** *p < 0.05, **p < 0.01.

Because of the different immunogenicity, peptides with the ability to stabilize HLA-A*0201 molecules are not always able to induce tumor-specific CTLs. Therefore, we used enzyme-linked immunospot assay (ELISPOT) to detect the ability of high affinity peptides identified in the T2 binding assays to induce CTLs to secrete IFN-γ. Specifically, PBMCs were isolated from healthy donors. Part of the cells were induced by cytokines to form DC and then were co-incubated with mixed high-affinity peptides. These peptide-loaded DCs were then co-cultured with the rest of the PBMCs to induce peptide-specific CTLs. In the ELISPOT experiment, we found that numbers of interferon-γ-secreting CTLs induced by different peptide-loaded DCs differed a lot. The positive control CMV peptide induced more than 400 spots per well. Among the 7 candidate peptides, peptide No. 8 (VLMLIQKLL), followed by peptide No. 5, induced the highest number of interferon-γ-secreting CTLs (**Figures 3A, B**). The induction of interferon-γ by the other five peptides was mild (**Figure 3B**). In order to further verify the specificity of interferon-γ secretion, we also used peptide No. 1 (as irrelevant control, **Figure S2A**) and peptide No. 8 (**Figure S2B**) as antigens for CTLs. As expected, peptide No. 8 produced more spots (**Figure S2C**). It is worth mentioning that no spots were observed when PBMCs of healthy donors and MM patients were directly stimulated with peptide No. 8 (**Figures S2D, E**). These results suggest that peptide No. 8 may be a natural antigen peptide of AKAP4.

### CTLs induced by peptide No. 8 can specifically lyse AKAP4^+^ myeloma cell line *in vitro*

**Figure 4:**
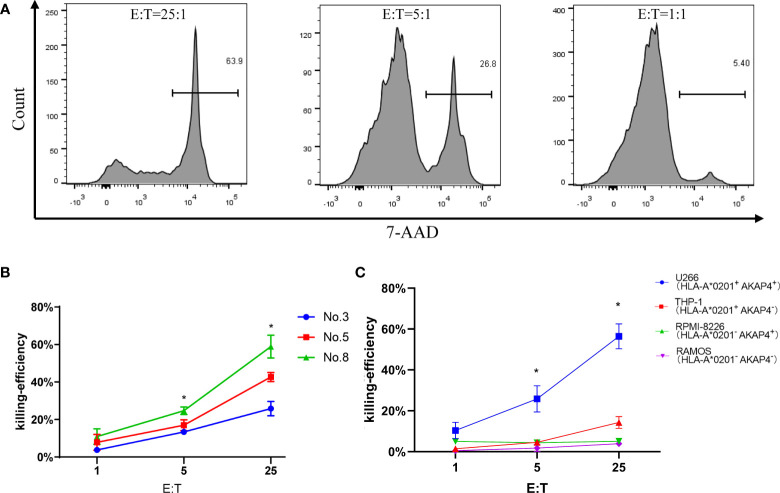
Tumor cell line killing assay shows the anti-myeloma effect of peptide No. 8 specific CTLs in vitro. **(A)** The killing efficiency of CTL stimulated by No. 8 antigen peptide on U266 cell line under different effect target ratio (the effective target ratio is 25 in the left figure, 5 in the middle figure, and 1 in the right figure). **(B)** Compared to CTLs induced by peptide No. 5 and No. 3, peptide No. 8 specific CTLs lyse U266 cell more efficiently. **(C)** Peptide No. 8 specific CTLs lyse HLA-A*0201^+^ AKAP4^+^ myeloma cell line U266, while the other HLA-A*0201^-^ or AKAP4^-^ cell line is not killed efficiently (summarized the results of three independent experiments). Friedman test showed the *p* < 0.05 for U266 to other cell lines. Kruskal–Wallis rank sum test was performed to assess the significance in **C** *p < 0.05.

To further test whether peptide No. 8 could induce CTL-specific killing to myeloma cell lines, we carried out a tumor cell line killing assay. The killing efficiently is positively correlated with the effector-to-target (E:T) ratios, the specific killing ability to U266 cells reached ~64% at an E:T ratio of 25:1, while the killing efficiency was only about 27% and 5% at the E:T ratios of 5:1 and 1:1, respectively (**Figure 4A**). Among the three tested peptides, the CTLs induced by peptide No. 8 showed the highest killing ability compared to peptides No. 3 and No. 5 at all E:T ratios (**Figure 4B**). Interestingly, we found that the killing ability of CTLs to tumor cell lines depended on the expression of AKAP4 and HLA-A*0201. As shown in **Figure 4C**, the killing rates of CTLs to RPMI-8226 (AKAP4^+^ HLA-A*0201^-^), THP-1 (AKAP4^-^ HLA-A*0201^+^), and RAMOS (AKAP4^-^ HLA-A*0201^-^) cells were significantly lower than that to U266 cells, which are AKAP4^+^ and HLA-A*0201^+^.

### Tumor growth was inhibited by peptide No. 8 specific CTLs *in vivo*

**Figure 5:**
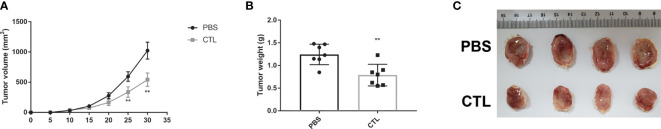
Tumor model showed the anti-myeloma effect of the peptide No. 8 specific CTLs in vivo. U266 cells (5 × 10^5^) were inoculated s.c. into NTG mice (day 0) followed by No. 8 peptide induced CTLs (5 × 10^6^) or PBS injected intravenously on day 1 and day 7. After the mice were killed in the end point of the experiment (Day 30), the tumors were peeled off, weighed, and photographed. Independent experiment was performed two times. The number of animals in the first experiment was 3 and the second was 4. The results of the two experiments were the same. The summarized results of the two experiments are shown in **(A)** (the tumor growth curve) and **(B)** (tumor weights). **(C)** The results of the second experiment (excised tumors). Two-way-ANOVA showed that the *p*-value is <0.001 for CTL and PBS in **(A)** Unpaired *t*-test was performed to assess the significance in **A** and **B** **p < 0.01.

Furthermore, to test whether peptide No. 8-induced CTLs have antitumor functions *in vivo*, we adopted the U266 graft tumor model. As shown in **Figure 5**, U266 tumor growth was suppressed (**Figure 5A**) and tumor weights (**Figure 5B**) were lower in the mice that received the peptide No. 8-induced CTL treatment than those that received PBS. A group of tumor pictures is shown in **Figure 5C**, indicating that the peptide No. 8-induced CTL is efficacious in inhibiting U266 HLA-A*0201+AKAP4+ myeloma tumor *in vivo*. We repeated two experiments independently, and the results of individual experiments are shown in **Figure S3**.

## Discussion

MM is still considered incurable, although the new chemotherapy regimens have greatly improved the prognosis. The high recurrence rate after treatment remains an unsolved problem. Immunotherapy, especially CAR-T therapy, is considered the most promising strategy against this malignant tumor due to the non-cross-resistant mechanisms of actions (20). However, even after CAR-T treatment, patients will eventually relapse. Therefore, the combination therapy against different targets might be a reasonable strategy. Some studies have made a breakthrough in the treatment of myeloma using the combination of natural killer cells, cytotoxic T lymphocytes, and T-cell receptor transgenic T (TCR-T) cells.

AKAP4 is mainly anchored in the tail of spermatozoa and can affect the intracellular environment and signal by regulating the PKC-ERK1/2-MAPK cascade (21). Its expression and localization in myeloma cells had been confirmed (17, 22), but accurate function of AKAP4 in the pathogenesis of MM has not been elucidated yet. Our study confirmed the expression of AKAP4 both in the cytoplasm and on the cell surface of myeloma cell lines and human primary myeloma cells. The intracellular expression of this protein is significantly higher than cell surface expression, suggesting that CTLs or TCR-T strategies would be more effective than monoclonal antibodies or CAR-T therapy (23). Given that CTAs are usually expressed in normal testicular tissue, the “on-target, off-tumor” effect should be considered when planning immunotherapy against AKAP4. Fortunately, since most MM patients are diagnosed at old age, with a median age of diagnosis at ~67 years old (20), their requirements for children are likely minimal. Therefore, AKAP4 may be a good target for MM immunotherapy.

Our study confirmed the high expression of AKAP4 in cytoplasm and low expression on cell surface in both primary myeloma cells and myeloma cell lines. However, we noticed that CTL against AKAP4 is almost absent in the patients (**Figures S2D–F**), despite the high expression of AKAP4 in the primary myeloma cells. This can be partially explained by the fact that tumor antigens are much less antigenic than viral antigens (17). Additionally, the multiple tumor immune escape pathways and the fragile immune system in patients with MM could have contributed to this scenario. In our study, we found that some high-affinity peptides in the T2 binding assay were not able to induce CTLs to secrete INF-γ, suggesting that high affinity does not stand for high immunogenicity. It is possible that the antigenic epitopes of AKAP4 were not well presented to CTLs *in vivo*, which also contributes to the nearly absent AKAP4-specific CTLs in patients. However, better outcomes and even the possibility of cure can be achieved after the patients have acquired tumor-specific immune response through antigenic vaccines or TCR-T technology, especially after reducing the tumor load of myeloma patients to a certain level using chemotherapy first.

Compared to other myeloma antigenic peptide epitopes that have been published, such as the anti-FcRH5/CD3, in which the peptide-induced CTLs had 85% killing efficiency *in vitro* against target cell lines at an E:T ratio of 10:1 (24), and two other weaker ones, XBP1 (25) and CS1 (26), which induced a CTL killing efficiency of about 50% against myeloma cell lines at an E:T ratio of 10:1, the APAK4 antigenic peptide-induced CTLs in our study had lower killing efficiency (27%–60%). Possible reasons include the following: First, unlike from the reported studies, the effector cells used for the killing assay in our study were mixed types of lymphocytes, of which the proportion of CD8^+^ T cells could only reach about 30% by the time of the killing assay; thus, the true E:T ratio would be much lower than the ratios reported. Second, the main killing validation method used in our study was the flow cytometry, which may not be as sensitive as other experimental methods such as ^51^Cr release assay for determining killing ability. Third, although the dendritic cells are the most potent antigen-presenting cells found *in vivo* (27), the DCs used in our study were induced from PBMCs *in vitro*, which may not be able to fully activate CTLs and induce cytotoxicity.

In summary, our study confirmed the high expression of AKAP4 in cytoplasm and low expression on cell surface in both primary myeloma cells and myeloma cell lines. We have screened 12 potential antigenic peptide epitopes and identified seven high-affinity peptides that can bind HLA-A*0201 molecules in the T2 binding assay, of which only two peptides were able to induce the specific CTLs to secrete IFN-γ when encountering the cognate peptides. AKAP4 630–638 (VLMLIQKLL), which showed the strongest immunogenicity, could induce specific CTLs that showed significant anti-myeloma activity both *in vitro* and *in vivo*. The specific lysis of the AKAP4^+^ HLA-A*0201^+^ MM cell line provided strong evidence that AKAP4 630–638 (VLMLIQKLL) is a real antigenic epitope. Therefore, it is promising that the VLMLIQKLL epitope can be used as another myeloma antigen peptide to develop DC vaccine (28), TCR-T (29, 30), TCR-mimic antibody (31), or T-cell bispecific antibody (32) against myeloma.

## Data availability statement

The original contributions presented in the study are included in the article/**Supplementary Material**. Further inquiries can be directed to the corresponding authors.

## Ethics statement

The studies involving human participants were reviewed and approved by Committee of Peking University First Hospital. The patients/participants provided their written informed consent to participate in this study. The animal study was reviewed and approved by Ethics Committee of Peking University First Hospital.

## Author contributions

NM and HL contributed to data collection, data analysis, and preparation of the manuscript. YZ, WL, ZL, QW, YS, LW, and HR contributed to data collection. HL and YD contributed to the research design and preparation of the manuscript and reviewed the manuscript. All authors contributed to the article and approved the submitted version.

## Funding

This article was supported by Beijing Natural Sciences Foundation (Grant No. 7192205).

## Conflict of interest

The authors declare that the research was conducted in the absence of any commercial or financial relationships that could be construed as a potential conflict of interest.

## Publisher’s note

All claims expressed in this article are solely those of the authors and do not necessarily represent those of their affiliated organizations, or those of the publisher, the editors and the reviewers. Any product that may be evaluated in this article, or claim that may be made by its manufacturer, is not guaranteed or endorsed by the publisher.
