# Supplementary material for: Identification of CD8+ T-cell epitope from multiple myeloma-specific antigen AKAP4
Source: Front Immunol. 2022 Jul 28;13:927804. doi: 10.3389/fimmu.2022.927804 (PMC9366082; doi:10.3389/fimmu.2022.927804)
Supplement: Supplementary file 1 [file DataSheet_1.docx]

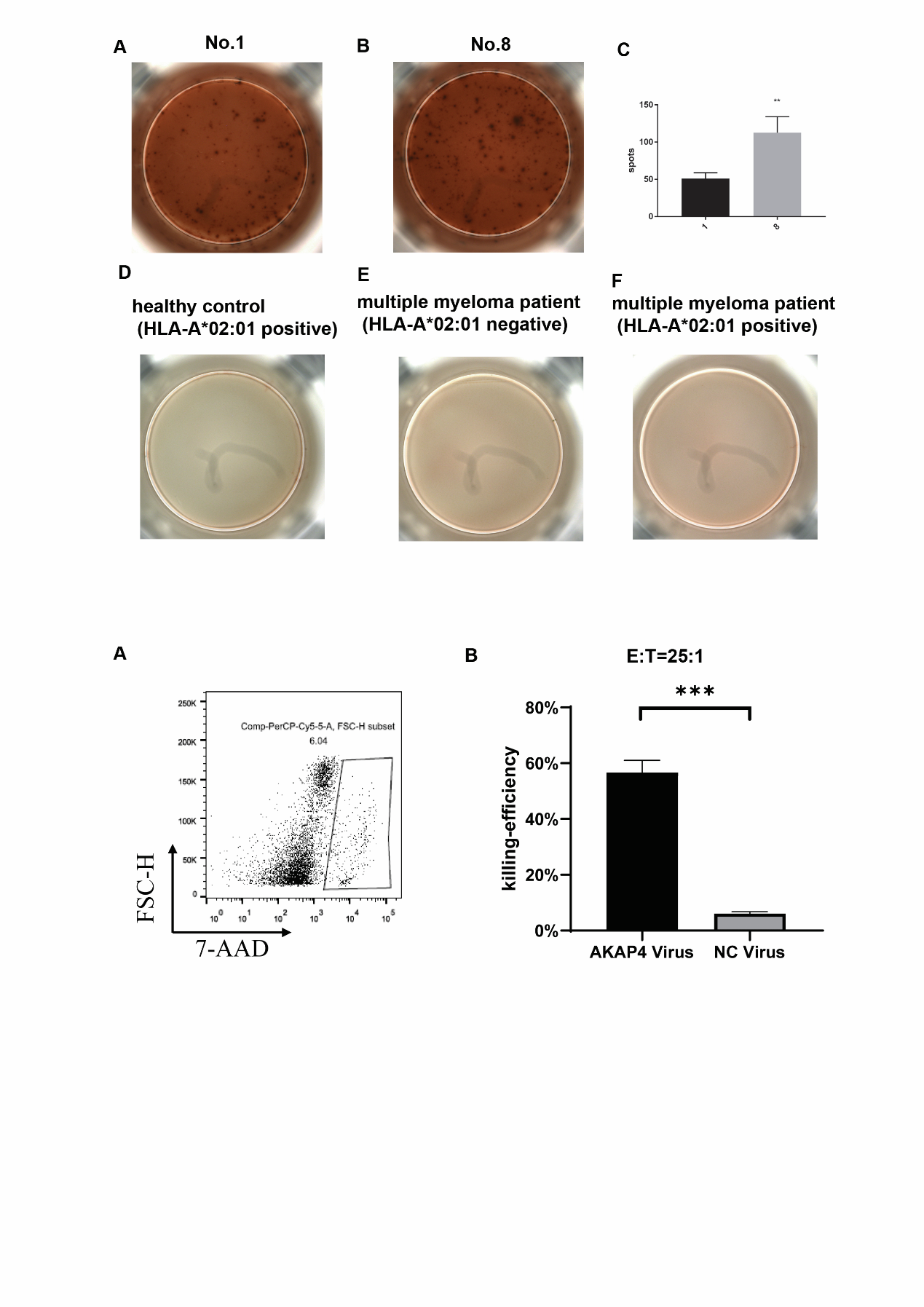


**Figure S1**

**The killing efficiency of CTLs induced by AKAP4 adenovirus-transfected DC on U266 cells. (A)** The killing effect of CTLs induced by DC cells which transfected by negative control virus (NC, Empty adenovirus vector without *AKAP4*); **(B)** Statistical difference of the killing efficiency of CTLs induced by AKAP4 adenovirus-transfected DC and NC virus-transfected DC (Summarized the results of three independent experiments). ***P < 0.001.


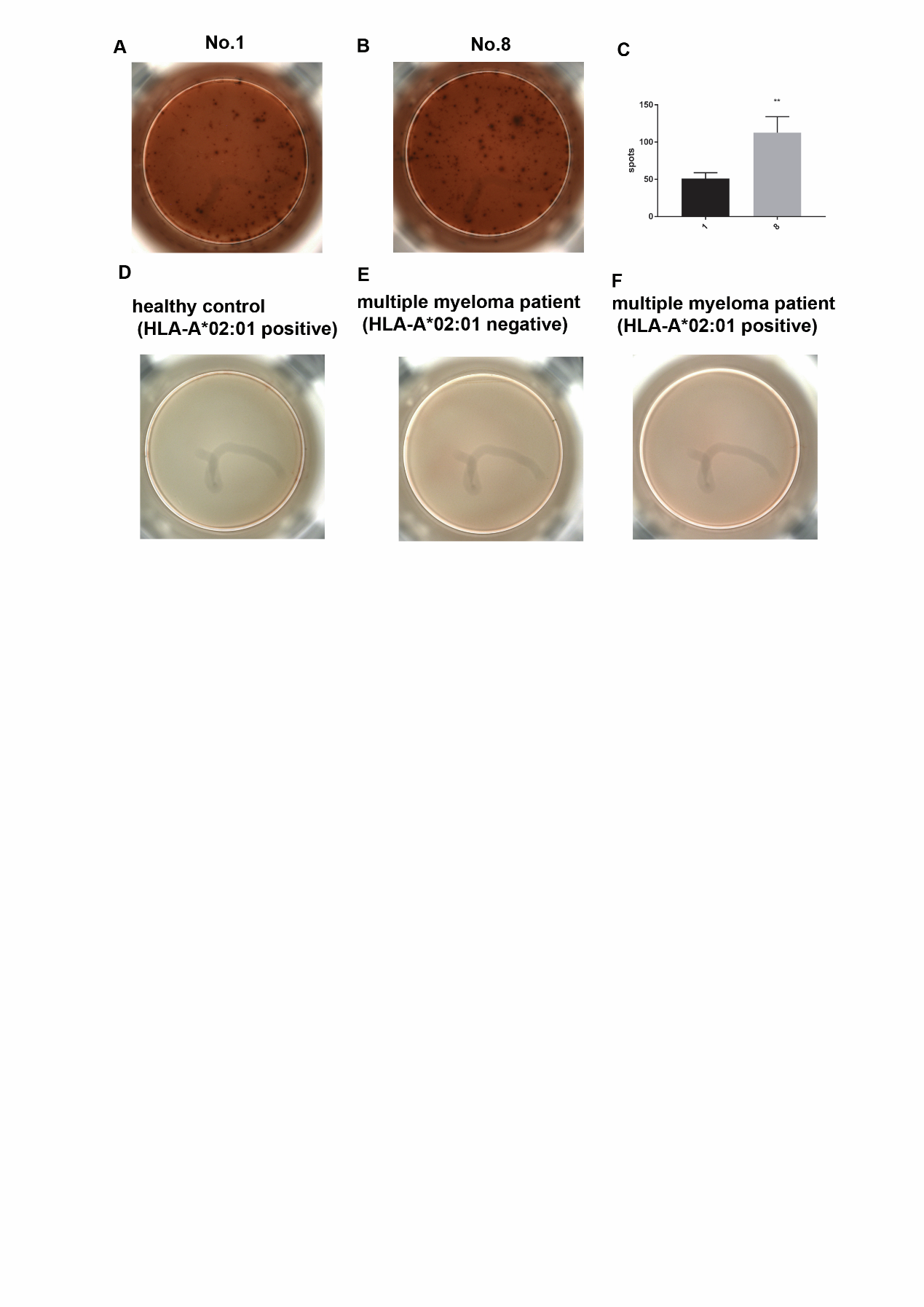


**Figure S2**

**ELISPOT assay of IFN-γ secretion. (A)** CTLs induced by peptide No.1 (as irrelevant control), **(B)** CTLs induced by peptide No.8, **(C)** Statistical difference between the two groups (Summarized the results of three independent experiments). **P < 0.01. **(D-F)** PBMC of healthy donors and MM patients were directly stimulated with peptide No.8.


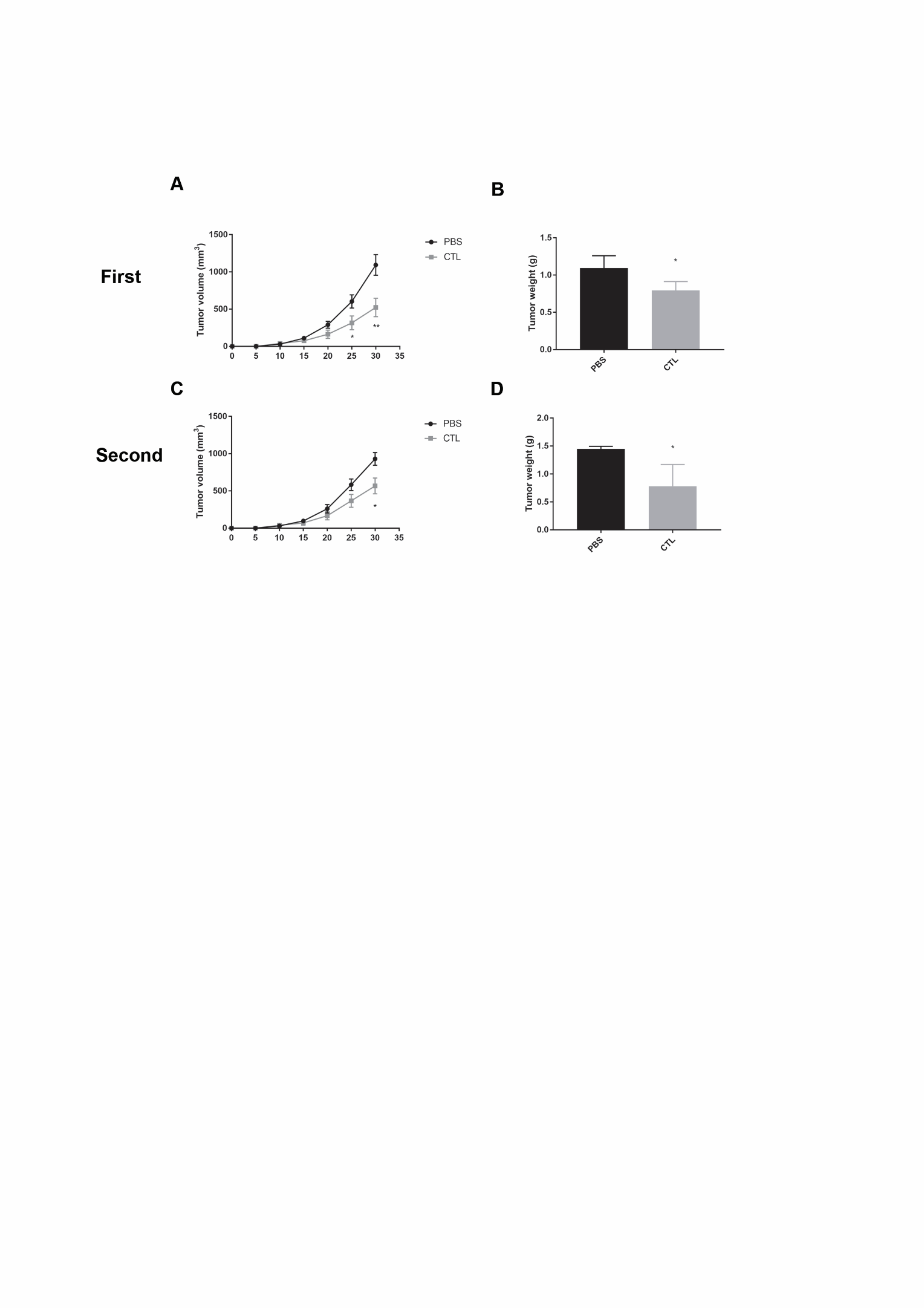


**Figure S3**

**Tumor Model showed the anti-myeloma effect of the peptide No.8 specific CTLs *in vivo*.** U266 cells (5× 10^5) were inoculated s.c. into NTG mice (day 0) followed by No. 8 peptide induced CTLs (5× 10^6) or PBS injected intravenously on day 1 and day 7. After the mice were killed in the end point of the experiment (Day 30), the tumors were peeled off, weighed and photographed. Independent experiment was performed 2 times. The number of animals in the first experiment was 3 **(A, B)** and the second was 4 **(C, D)**. **(A, C)** the tumor growth curve; **(B, D)** tumor weights. Two-way-ANOVA showed the P value is < 0.001 for CTL and PBS in A and C. Unpaired T test was performed to assess the significance. *P < 0.05, **P < 0.01.
